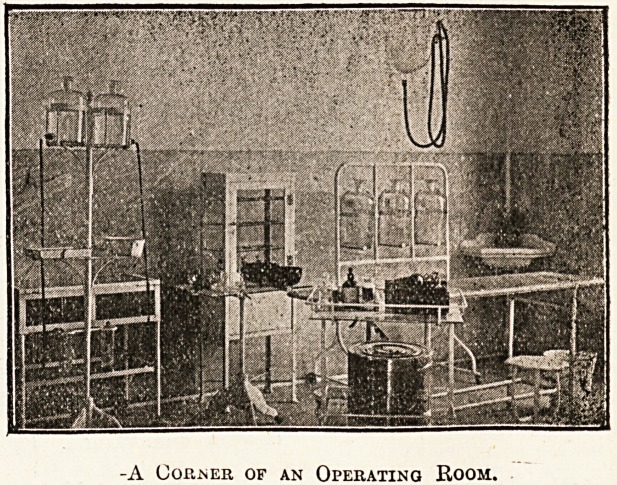# Building a Hospital in India

**Published:** 1915-04-24

**Authors:** C. H. Buck

**Affiliations:** Deputy Commissioner.


### BUILDING A HOSPITAL IN INDIA.

### II.—The Story of the Karnal District Hospital.*

By Major C. H. BUCK, I.A., Deputy Commissioner.

### The Workman's Point of View.

It is surprising what a variety of workmen are required for building in India; there seems also to be no end of them, and it is seldom that one man will do another's job. Thus, one man fetches water, he pours it into a receptacle alongside the mason, and another person, perhaps this gentleman's tiny son, sprinkles it over the masonry; one workman carries the lime, another the brick-dust, a third mixes the mortar, a fourth carries it to the mason, and so on. Soil for bricks is excavated by one set of men, carried by another, moulded by other persons, carried probably on minute donkeys, and placed in the kiln by yet others. The bullock carts convey materials to the site, but the cartmen, instead of assisting to load and unload the conveyances, smoke *hookahs* while that is being done.

The scaffolding is erected by one lot of labourers, but experts are called in to tie the lashings—which is perhaps just as well. When the walls get high it is laughable to see how the bricks go up; each ladder has its complement of labourers—if so they can be termed—who may be boys, or even girls; the bricks are passed up from hand to hand until they reach the top, where more individuals deal them out to the bricklayers. I have often watched the persons working at a single small room, and been amused to see thirteen all doing nothing, while the fourteenth takes five minutes to lay one or two bricks; he then pauses for rest and a pull at the *hookah*, which is very much *en évidence*, one pipe doing duty for half a dozen, who frequently stop work, squat down, and take their turn at a whiff.

### Noises and Bribery.

And what a din there is throughout the day! Besides the ordinary noises of building to which one is accustomed in England, there are, in India, donkeys which bray, dogs which howl, tame birds which chirp (many masons keep a partridge or a *chikor* in a cage alongside them while working), the whacking of bullocks, and, last but not least, the continual jabber and run of good-natured chaff,

King Edward VII. Memorial Hospital, Karnal.

P. Wards = Private Wards. F. Ward = Female Ward. OP. Rooms = Operating Rooms. Admin. = Administration. Lav. = Lavatories. Disp. = Dispensary.

One of the Wards. Each contains Twenty Patients.

* The first article appeared on April 17.

with its accompaniment of foul oaths which occasionally end in a battle royal of shrieking, cursing, and yelling, but very seldom in blows.

THE DISTRIBUTION OF SWEETMEATS.

It is a wonder that any building is ever finished in Asia, but there are ways and means of getting things done; one of these is the distribution of sweetmeats at intervals to all employed when they have worked quickly; another is a competition between two or more parties for a reward to the one which finishes certain work first.

The construction of the water-works was rather a business. First of all, a large well was sunk,

BUILDING THE OPERATING ROOMS AND, BEYOND, ONE OF THE MALE WARDS.

a plentiful supply of fresh water being found only twenty-five feet below the surface; two large iron tanks were erected close by, and a pump and a 5-h.p. oil engine fitted into a shed alongside. All the materials for these had to be obtained from a firm at Calcutta, over a thousand miles away, and, when the pump would not work, an expert had to be obtained from there to set it right.

THE EXPERT AND THE LOCAL PLUMBER.

However, when the expert actually arrived he failed in his aim after two weeks' hard work; and finally the firm had to supply another pump. When water was at length pumped into the tanks, a wretched sub-overseer, strictly against orders, started the water down the pipes without opening the taps, and thereby caused numerous bursts and much further delay, for fresh pipes had to be obtained from the other extremity of India. Good people in England send word to the local plumber when a tap gets broken or worn out, and he supplies a new one and fixes it on the following day; in India one has to take the measurements, look up a suitable tap in a catalogue, and order it from a firm some hundreds of miles away, perhaps to find they have run out of stock of that particular article.

The furniture for the hospital was obtained partly from England, but mostly from Multan, in the Punjab, where a Parsee firm manufactures quite high-class enamelled ware. The surgical instruments were all obtained from England.

THE "EN ECHELON" PLAN.

One of the noticeable features of the hospital is that all the accommodation is on the ground floor, so that staircases are not required except in the towers where the drug stores are kept. The principal group of buildings consists of an administration block in the centre with two male wards on either side *en échelon*; immediately in rear is a building containing two large operating rooms, with anæsthetic, sterilising, and instrument rooms; all of these are surrounded with wide verandahs and connected by broad cloisters.

THE STAFF QUARTERS.

To the north is the female portion of the hospital in a self-contained group. There are numerous quarters for the staff and servants, while the sanitary arrangements, which are very complete, include a special place for washing clothes and a large roofed cage, in which the dirty clothes of fresh admissions are exposed on shelves to the sun and air before being handed over to be washed, ticketed, and stored. Many of the patients fail to recognise their clothing when they receive it again on discharge!

The hospital accommodates 120 patients and has an Indian staff of one assistant surgeon and his sub-assistant, a female sub-assistant surgeon, and a number of nurses, dressers, compounders, and ward servants, besides a full staff of menials and a mechanic in charge of the water-works. The whole institution is under the direct control of the civil surgeon of the district, who is usually a European.

STATUE OF KING EDWARD.

One of the inscriptions in the entrance hall of the main building records that this hospital was

A CORNER OF AN OPERATING ROOM.

erected in memory of the King-Emperor Edward VII., who at all times exerted himself to secure the advance of medical science, the allayment of suffering, and the general welfare of his subjects. In front of this stands a fine bronze bust of his late Majesty.

## Figures and Tables

**Figure f1:**
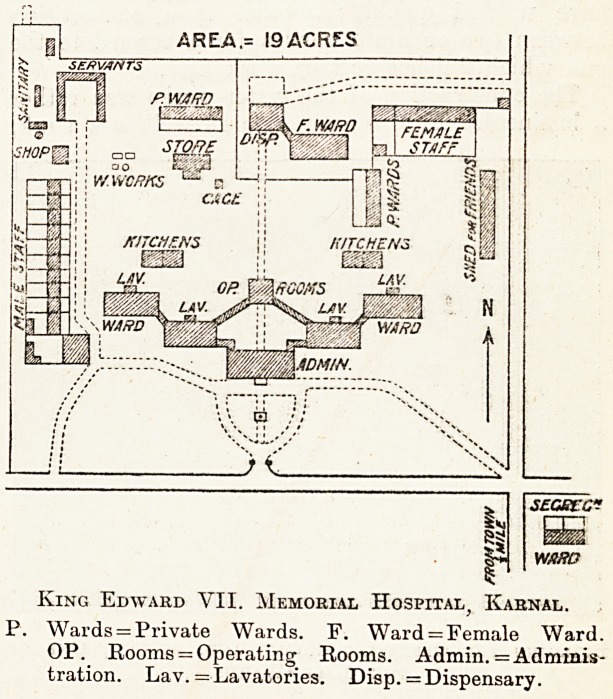


**Figure f2:**
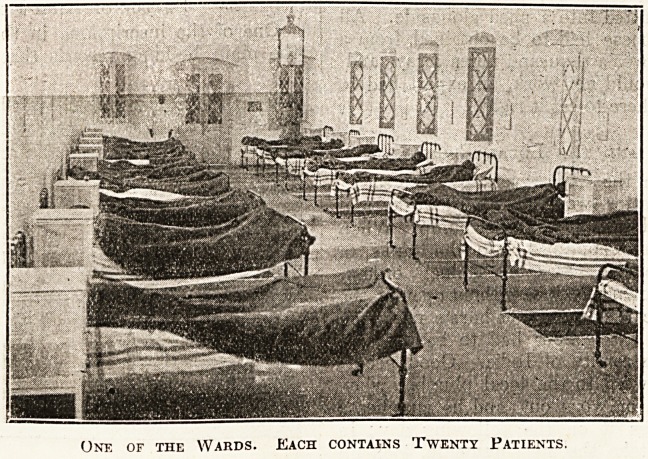


**Figure f3:**
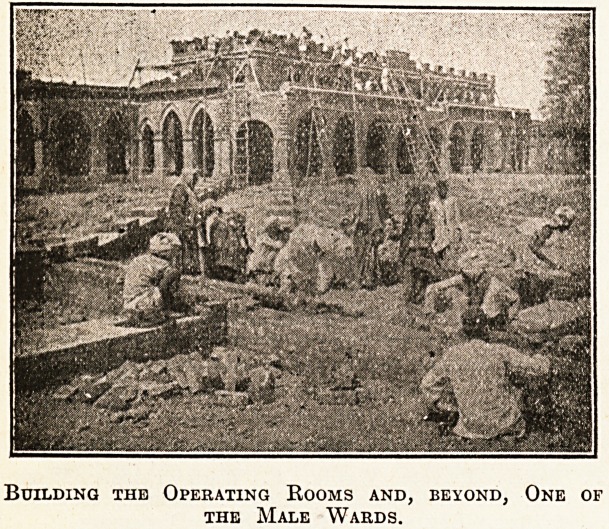


**Figure f4:**